# Resident- and Facility-Level Predictors of Resident-Reported Quality of Life in Assisted Living

**DOI:** 10.1093/geroni/igaf122.705

**Published:** 2025-12-31

**Authors:** Tetyana Shippee, Romil Parikh, Molin Ji, Timothy Beebe, Mark Woodhouse

**Affiliations:** University of Minnesota, Minneapolis, Minnesota, United States; University of Minnesota School of Public Health, Minneapolis, Minnesota, United States; University of Minnesota, Minneapolis, Minnesota, United States; University of Minnesota, Minneapolis, Minnesota, United States; University of Minnesota, Minneapolis, Minnesota, United States

## Abstract

Ensuring high-quality care in assisted living (AL) is a growing priority, yet little is known about factors shaping resident-reported quality of life (QoL). Minnesota recently implemented statewide measurement and public reporting of QoL, providing a unique opportunity to examine predictors of resident experiences. This study evaluated resident- and facility-level factors associated with seven QoL domains: staff, environment, food, engagement, autonomy, culture, and security. We analyzed cross-sectional data from 11,684 AL residents in facilities with more than five beds who participated in the 2024 state QoL survey (90% aged ≥65 years, 66% female, 84% White). Using multivariable linear mixed regression with a facility-level random intercept, we accounted for facility-level clustering. Mean QoL scores ranged from 1.6 to 1.9 out of 2, with food and engagement rated lowest and environment highest. Older residents reported higher scores across most QoL domains (β, 0.02–0.08; p < 0.05). Women rated food and security lower (β, -0.04 and -0.02; p < 0.05), while American Indian/Alaskan Native residents reported lower scores in culture, security, staff, and environment (β, -0.05 to -0.10; p < 0.05). Smaller facilities (< 25 beds) had higher scores in food, staff, and autonomy (β, 0.02–0.07; p < 0.05). Facilities with dementia care unit licensure had higher food scores but lower autonomy, staff, and security scores (β, -0.02 to -0.03; p < 0.05). Findings highlight disparities in resident QoL, underscoring the need for targeted interventions and policy efforts to enhance resident well-being in AL.

